# Laminin β2 Chain Regulates Cell Cycle Dynamics in the Developing Retina

**DOI:** 10.3389/fcell.2021.802593

**Published:** 2022-01-12

**Authors:** Dmitri Serjanov, Galina Bachay, Dale D. Hunter, William J. Brunken

**Affiliations:** Department of Ophthalmology and Visual Sciences, Upstate Medical University, Syracuse, NY, United States

**Keywords:** extracellular matrix, laminin, dystroglycan (DG), cell cycle, retinal progenitor cell (RPC), retinal development

## Abstract

Vertebrate retinal development follows a highly stereotyped pattern, in which the retinal progenitor cells (RPCs) give rise to all retinal types in a conserved temporal sequence. Ensuring the proper control over RPC cell cycle exit and re-entry is, therefore, crucially important for the generation of properly functioning retina. In this study, we demonstrate that laminins, indispensible ECM components, at the retinal surface, regulate the mechanisms determining whether RPCs generate proliferative or post-mitotic progeny. *In vivo* deletion of laminin β2 in mice resulted in disturbing the RPC cell cycle dynamics, and premature cell cycle exit. Specifically, the RPC S-phase is shortened, with increased numbers of cells present in its late stages. This is followed by an accelerated G2-phase, leading to faster M-phase entry. Finally, the M-phase is extended, with RPCs dwelling longer in prophase. Addition of exogenous β2-containing laminins to laminin β2-deficient retinal explants restored the appropriate RPC cell cycle dynamics, as well as S and M-phase progression, leading to proper cell cycle re-entry. Moreover, we show that disruption of dystroglycan, a laminin receptor, phenocopies the laminin β2 deletion cell cycle phenotype. Together, our findings suggest that dystroglycan-mediated ECM signaling plays a critical role in regulating the RPC cell cycle dynamics, and the ensuing cell fate decisions.

## Introduction

The retina is a highly structured portion of the central nervous system (CNS). During vertebrate retinal development, retinal progenitor cells (RPCs) give rise to all retinal cell types in a conserved temporal sequence. With each cell cycle, a subpopulation of RPCs leaves the cells cycle to become retinal neurons. The first retinal cells to exit the cell cycle are ganglion cells, followed by overlapping waves of differentiating horizontal cells, amacrine cells, cone photoreceptors, rod photoreceptors, bipolar cells, and Müller glia (R. W. Young, 1985; Turner and Cepko, 1987; Holt et al., 1988; Turner et al., 1990). The balance between RPC self-renewal and differentiation is of great importance to ensure the proper development and organization of the retina and this orderly array of cell fates.

**GRAPHICAL ABSTRACT:**
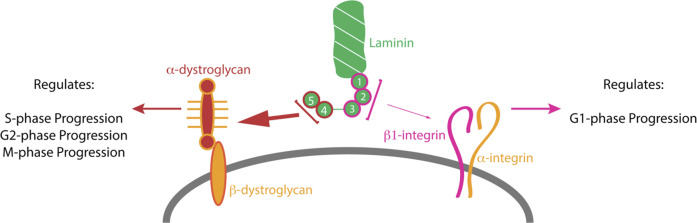


The RPC fate choice is tightly regulated by a number of intrinsic and extrinsic cues. Mitotic spindle orientation has been strongly linked to cell fate in various systems (Huttner and Kosodo, 2005; Morin and Bellaiche, 2011). We have previously reported that β2-containing laminins modulate the RPC fate by modulating their mitotic axis (Serjanov et al., 2018). However, mitotic spindle orientation is not the only factor regulating cell fate decisions. Cell cycle dynamics have been shown to be of crucial importance in governing the cell fate determination in CNS progenitors (Calegari, 2003; Calegari, 2005; Baye and Link, 2007a; Baye and Link, 2007b; Pilaz et al., 2009). The cell cycle consists of four distinct phases. DNA duplication occurs during the synthesis phase (S-phase), and separation of duplicate chromosomes between two daughter cells occurs in mitosis (M-phase). S and M-phases are separated by two gap phases—G1 and G2. G1 occurs between mitosis and the succeeding S-phase, while G2 lies between S and M-phases. Earlier studies noted extended G1 duration of the radial glia (RG) correlated with the timing of neurogenesis (Calegari, 2003; Calegari, 2005; Baye and Link, 2007a; Baye and Link, 2007b; Pilaz et al., 2009), suggesting that cell cycle timing plays a role in cell fate determination. A later study determined that G1 extension is associated with the restricted intermediate progenitor cells (IPCs), and that the observed progenitor population-wide G1 lengthening is associated with the increased presence of these cells (Arai et al., 2011). Both RG and IPCs that underwent terminal division displayed shortened S-phase length with cells presumably spending less time error checking. Extended mitosis duration has also been observed in conditions associated with premature progenitor differentiation such as lissencephaly and microcephaly (Pilaz et al., 2016; Bershteyn et al., 2017).

Various ECM components such as collagens (Koohestani et al., 2013) and laminins (Domogatskaya et al., 2008), as well as their receptors (Clements et al., 2017), have been shown to affect cell proliferation, though the exact mechanisms of these interactions remain relatively unknown. A link between ECM rigidity and cell cycle regulators had been noted previously (Gerard and Goldbeter, 2014). Taken together, all these data suggest that ECM regulates cell cycle dynamics as well as cell fate via a combination of molecular signaling and biophysical interactions with cells. Previous studies demonstrated that laminins, which are indispensable components of the basement membrane assembly, play important roles throughout retinal development. Laminins, heterotrimeric proteins, containing an α, a β and a γ chain are produced by the retinal neural epithelium early in development and then later by Müller cells (Libby et al., 1997). β2-containing laminins are indispensable for the formation of the inner limiting membrane (ILM) but are not for other retinal basement membranes such as vascular and Bruch’s (Pinzón-Duarte et al., 2010). β2-containing laminins have been shown to play a role in the development of rod and bipolar cell production (Hunter et al., 1992; Hunter and Brunken, 1997). Genetic ablation of the laminin β2 subunit results in a host of retinal developmental abnormalities including: retinal dysplasia (Pinzón-Duarte et al., 2010); photoreceptor synapse malformation and instability (Libby et al., 1999; Hunter et al., 2019); dysgenesis of dopaminergic amacrine cells (Denés et al., 2007); and vascular development (Biswas et al., 2017; Biswas et al., 2018).

In particular, β2-containing laminins in the ILM are critical components for a wide variety of cell-matrix interactions. β2-containing laminins were identified as substrates for integrin-mediated astrocyte migration (Gnanaguru et al., 2013) as well an attachment site for Müller cells thereby providing polarity cue for the normal distribution of aquaporin channels (Hirrlinger et al., 2011). Moreover, *Lamb2* deletion resulted in the loss of basal processes from RPCs, producing an IPCs-like morphology with disruptions in the cytokinesis and a premature cell cycle exit with a concomitant overproduction of rods at the expenses of later born cell types (Serjanov et al., 2018). Because of the critical role ILM laminins play in cellular processes of cells adherent to it, we investigated the effects of β2-containing laminins on the RPC cell cycle dynamics.

In this study, we determined the cell cycle dynamics of the RPCs in postnatal WT mouse retina, and compared them with those of the *Lamb2* ^*−/−*^ animals *in vivo*. Here, we show that deletion of laminin β2 results in a substantial decrease of the RPC S and G2-phase lengths, as well as extended M-phase durations. Ultimately, these changes result in an increased rate of cell cycle exit. We further analyzed the effects of β2-containing laminins on RPC cell cycle using the organotypic retinal culture approach, and showed that addition of exogenous β2-containing laminin to the retinal surface *ex vivo* rescues the cell cycle dynamics. Furthermore, we identified the laminin receptor dystroglycan (DG) as the receptor mediating the ECM-RPC signaling responsible for the observed cell cycle changes. Our data suggest a mechanism in which ECM contact is of key importance in regulating RPC cell cycle progression and the ensuing fate choice.

## Methods

### Antibodies

Phospho-Histone H3 (pSer28) (Sigma-Aldrich, Cat# H9908 RRID:AB_260096), Ki67 (BD Pharmigen, Cat# 550609), α-Dystroglycan blocking antibody (Ervasti et al., 1990; Ervasti and Campbell, 1991) Kevin Campbell, HHMI, University of Iowa, IIH6), β-1 Integrin blocking antibody (BD Biosciences, Cat# 553715 RRID:AB_395001), IgM Isotype Control from murine myeloma (Sigma-Aldrich, Cat# M5909 RRID:AB_1163655), Rat IgG2ak (BD Biosciences, Cat# 559073 RRID:AB_479682).

### Chemicals, Peptides, and Recombinant Proteins

EdU (Life Technologies, Cat# C10337), Hoechst (Invitrogen, Cat# H3570), Laminin-521 (BioLamina, Cat# LN521-3), and Donkey Serum (Sigma-Aldrich, Cat# D9663).

### Experimental Organisms

C57Bl6/J Mice (Jackson Laboratories, Bar Harbor ME, United States, RRID:IMSR_JAX:000664), *Lamb2* ^*−/−*^ Mice (Noakes et al., 1995).

### Software

Volocity 3D Image Analysis Software (Perkin Elmer, RRID:SCR_002668, SCR_002668), Graphpad Prism (Graphpad, RRID:SCR_002798, SCR_002798).

### Experimental Model

Deletion of the *Lamb2* gene and production of the *Lamb2* ^*−/−*^ mice have been described previously (Noakes et al., 1995). *Lamb2* ^*−/−*^ animals have been backcrossed to C57BL/6J over nine generations. Animals were maintained as heterozygotes. All animal procedures were performed in accordance with the Institutional Committee (IACUC) and the Institutional Biosafety Committee.

### Immunostaining

The following primary antibodies were used: rat anti-phospho Histone H3 (1:3,000, Sigma-Aldrich, H9908), mouse anti-Ki67 (1:300, BD Pharmigen, Cat# 550609). The following secondary antibodies were used: donkey anti-mouse 488 (1:300), donkey anti-rat 594 (1:500) (Life Technologies). Hoechst (1:100,000, Invitrogen, H3570) was used to stain cell nuclei. EdU was detected per vendor’s instructions.

### Retinal Preparations

Radial sections were prepared by making an incision in the ora serrata, fixing the eyes in 4% paraformaldehyde (PFA) for 15 min, cryoprotected in 20% sucrose, and mounted in O.C.T. embedding medium. 12 μm sections were collected on microscope slides with a cryostat. Sections were washed in PBS and then blocked for 30 min at room temperature in 5% donkey serum in PBS with 0.3% Triton X-100. Following washing in PBS, sections were incubated with primary antibodies overnight at 4°C in 5% donkey serum in PBS with 0.01% Triton X-100 (25 μl per section). Following washing in PBS, sections were incubated with secondary antibodies for 4 h at room temperature. Following incubation with secondary antibodies, sections were washed and mounted in Vectashield mounting medium (Vector Laboratories, H-1000) and imaged. To ensure comparable regions of the retina were analyzed, all sections used were oriented nasal-temporally and traversed the optic nerve. For flat-mount retinal preparations, eyes were enucleated and an incision was made in the ora serrata. The eye was then fixed in 4% PFA in PBS at 4°C for 30 min. The cornea and lens were then removed, and the sclera was peeled off. Four radial cuts were made to flatten the retina, which was then transferred to a well of a 24-well plate with PBS. After washing in PBS, retinas were incubated overnight at 4°C in blocking solution (5% donkey serum in PBS with 0.3% Triton X-100). Next, retinas were incubated with primary antibodies in 300 μl solution of 5% donkey serum in PBS with 0.01% Triton X-100, at 4°C for 24 h, washed, and incubated with secondary antibodies in same solution overnight. Following washing, tissues were mounted in ProLong^®^ Gold Antifade Reagent (Life Technologies, P36930).

### *Ex vivo* Rescue and Receptor Blocking Experiments

Organotypic retinal cultures with RPE intact were prepared as described previously (Serjanov et al., 2018). For rescue studies: following the medium change after first 24 h in culture, 10 μl medium containing 50 pMol laminin-521 was placed on the retinal surface. Medium containing no laminin was used as negative control. For receptor blocking studies: following the medium change, 10 μl medium containing 1nMol α-DG blocking antibody or 500 pMol β1-integrin blocking antibody or both was placed on the retinal surface. Nonspecific IgM and IgG2ak were used as isotype controls, respectively. After 3 days *in vitro*, cultures were fixed for flat-mounts or cryosections as described above.

### Cumulative S-phase EdU Labeling

*In vivo* cumulative S-phase labeling was performed by administering intraperitoneal injections of EdU in sterile saline at 3 h intervals, up to 33 h, at a dose of 100 mg/kg. Mice were collected 30 min following the last injection, and retinal preparations were performed as described above. To ensure consistent result, and control for possible circadian changes of cell cycle dynamics, all mine were collected at 11am at P3. *Ex vivo* cumulative S-phase labeling was performed by adding medium containing 2 μM EdU to the top and bottom compartments of the transwell inserts housing the retinal explants. The explants stayed in the labeling medium until being collected, for up to 21.5 h, prior to being collected and analyzed. To ensure consistent result, and control for possible circadian changes of cell cycle dynamics, all explants were collected at 11am of 3DIV.

### Percentage of Labeled Mitoses Studies

*In vivo* labeled mitoses studies were performed by administering a single intraperitoneal injection of EdU in sterile saline at a dose of 100 mg/kg. Retinas were collected at intervals of 1, 1.5, 2, and 2.5 h following the injection. To ensure consistent result, and control for possible circadian changes of cell cycle dynamics, every mouse was collected at 11am at P3. *Ex vivo* labeled mitoses studies were performed by adding medium containing 2 μM EdU to the top and bottom compartments of the transwell inserts housing the retinal explants. The explants stayed in the labeling medium until being collected, 1, 1.5, 2, and 2.5 h prior to being collected and analyzed. To ensure consistent result, and control for possible circadian changes of cell cycle dynamics, all explants were collected at 11am of 3DIV.

### Analysis of Cell Cycle Parameters

T_C_ and T_S_ calculations were done as follows. Labeling indices (LIs) for each cumulative label time point (from LI_[0.5]_ to LI_[33.5]_, reflecting the cumulative time of EdU labeling in hours) were calculated as a percentage of EdU+ cells within the neuroblastic layer (NBL) from 12 μm retinal sections. This approach allows for a quantification of samples of varying size and thickness due to the data being normalized to the total number of cells within the NBL rather than the number of EdU+ cells alone. The data points were then plotted as LI vs time of cumulative label. T_C_ and T_S_ were calculated as described previously (Nowakowski et al., 1989), with modification. Briefly, the original method relies on assumption that LI increased linearly until reaching a plateau, while our data demonstrate that the saturation curve is clearly non-linear. A quadratic function was used to describe the LI rise phase instead. As there appeared to be two plateaus in the *in vivo* experiments, a combination of two quadratic functions, or a quartic function, was identified as the best-fit model. Growth fractions (GFs) were defined as the average of LI values lying on the plateau. T_C_-T_S_ points were determined mathematically by calculating the intercept between the cumulative labeling curve and the line defining GF.

T_G2_ and T_M_ were determined as follows. The percentages of labeled mitoses were calculated from 12 μm retinal cross-sections of samples collected at 1, 1.5, 2, and 2.5 h after a single EdU injection, as percentages of PH3+ cells that were also EdU+. The data were then plotted as percentage of labeled mitoses vs time after EdU pulse. T_G2_ was calculated as the intersect of the abscissa and the line connecting the first two time points, as it reflects the time when PH3+ cells first start becoming EdU+. T_M_ was calculated as the time when the line connecting the last two time points reached 100% label, as it reflects the time when EdU+PH3+ cells replace EdU-PH3+ cells.

T_G1_ was calculated by combining the data from the cumulative S-phase EdU labeling, and labeled mitoses studies, as the former allows calculation of T_C_ and T_S_, and the latter allows calculation of T_G2_ and T_M_. The following formula was used: T_G1_ = T_C_-T_S_-T_G2_-T_M_.

### Experimental Design and Statistical Analysis

Mice or retinal explants were collected following respective EdU course. Sex of the animals was not assessed. Retinal sections were imaged using OptiGrid structured illumination microscopy (Qioptiq Imaging Solutions, Advanced Imaging Concepts, Princeton NJ) from peripheral regions of three retinas per genotype or *ex vivo* condition per time point, on a Nikon Eclipse Ni microscope with 40X oil immersion, or 20X air objectives at room temperature. 60X oil immersion objective was used to obtain 0.2 μm-step z stacks of retinal flat-mounts for mitotic staging studies. All measurements were performed using Volocity (Perkin-Elmer, Waltham, MA, United States). Labeling indices, percentages of labeled mitoses, and mitosis staging counts were performed by manually counting cells of interest in retinal cross-sections. The data points were compared using Student’s t-test (for two-condition comparison) or ANOVAs with Bonferroni’s multiple comparisons test (three or more conditions). Data were represented as mean ± S.E.M. Line slope comparisons were performed using ANCOVA. All statistical analyses and graphical representations were performed using GraphPad Prism 6.0. In figures, significances are represented as follows: *p ≤ 0.05; **p ≤ 0.01; and ***p ≤ 0.001. Adobe Illustrator CS3 and Adobe Photoshop CS3 were used for non-quantitative image editing and arrangement, such as image rotation and figure composition.

## Results

### RPC Cell Cycle Dynamics Are Laminin Dependent

**FIGURE 1:**
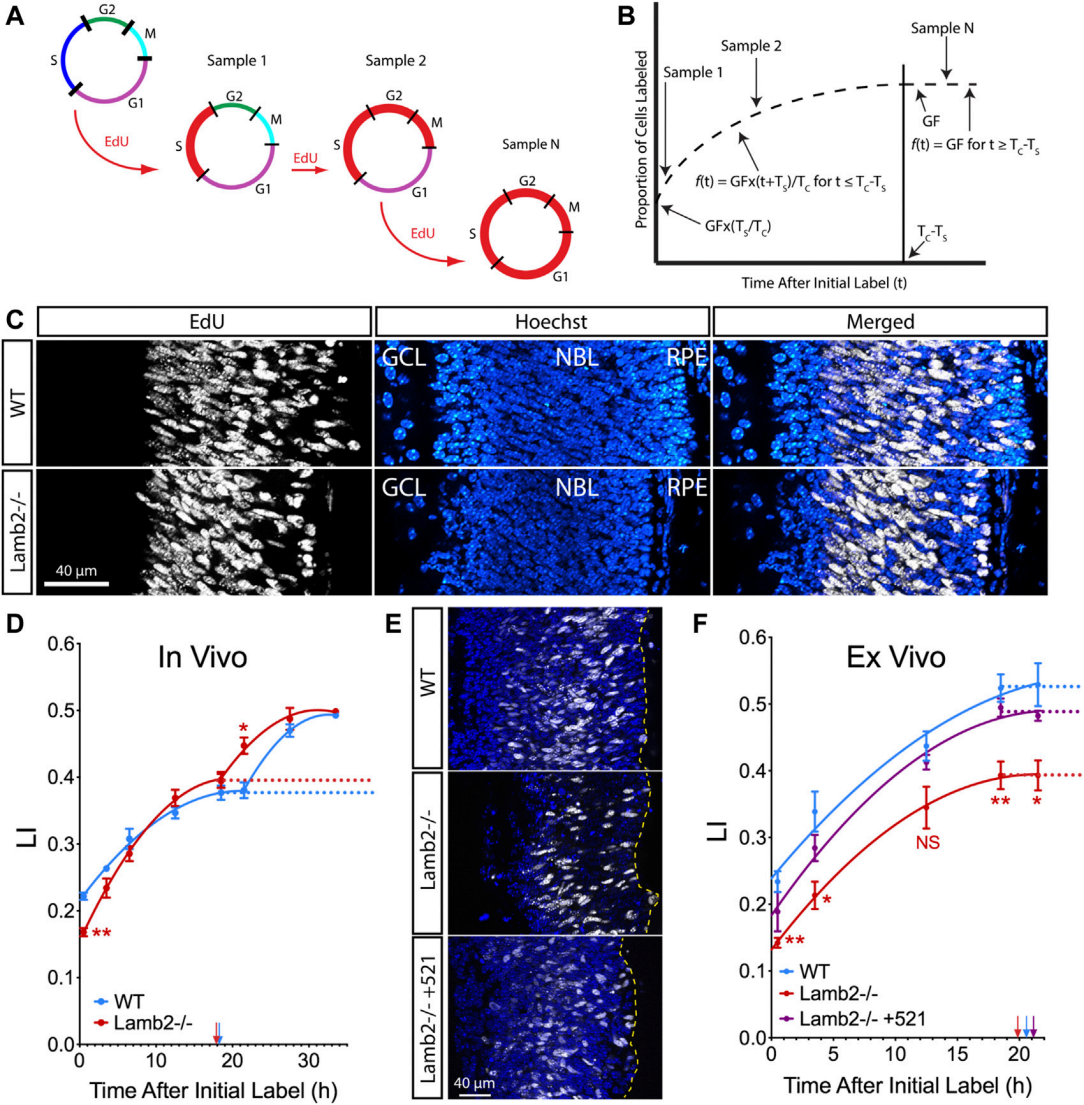
RPC T_C_ and T_S_ are laminin-dependent. **(A)**. Model illustrating the principle of cumulative S-phase labeling. This method relies on continuous EdU exposure, labeling successive populations of cells entering the S-phase. Red line marks the distribution of EdU-labeled cells within the cell cycle. **(B)**. Model illustrating the quantification of the results of cumulative EdU labeling. The proportion of EdU+ cells within the NBL was measured and plotted vs. the time off EdU exposure. A sharp initial rise in the labeling index reflects entry of unlabeled cells into the S-phase. This is followed by a plateau, which indicates the time when the entire proliferating population has been labeled. Growth fraction (GF) is measured as the ratio of EdU+ cells to total cell number in the NBL. T_C_–time of cell cycle. T_S_–time of the S-phase. **(C)**. Representative images of cross-sections of P3 retina that had been continuously labeled for 27.5 h. **(D)**. EdU saturation curves resulting from cumulative EdU labeling experiments in P3 retinas. **(E)**. Representative images of cross-sections of retinal explants that had been continuously labeled for 21.5 h. **(F)**. EdU saturation curves resulting from cumulative EdU labeling experiments performed on retinal explants. Dotted lines designate the GF at plateaus in the curves. Arrows indicate time when labeling index reached the plateau (T_C_-T_S_).

To determine whether RPC cell cycle progression is affected by the ILM composition, we examined the cell cycle dynamics using cumulative S-phase labeling with 5-ethynyl-2-deoxyuridine (EdU). P3 animals received consecutive intraperitoneal EdU injections at 3-h intervals to sequentially label cells in the S-phase, with an injection 30 min prior to tissue harvest (Figure 1A). Number of EdU+ cells within the neuroblastic layer (NBL) increases with time, until reaching a plateau at the maximum labeling index (LI), allowing determination of the growth fraction (GF—proliferating cell population relative to total cells in the tissue). Studying the increase and saturation of EdU+ population allows determination of lengths of the cell cycle (T_C_) as well as the S-phase (T_S_) (Figure 1B) (Nowakowski et al., 1989). LIs for each time point were calculated as percentages of EdU+ nuclei in the NBL (Figure 1C). Curiously, the saturation curves for both WT and *Lamb2* ^*−/−*^ retinas displayed a clear biphasic shape, with two rise-phases and plateaus, suggesting distinct waves of cell cycle exit and re-entry (Figure 1D). While the GFs were not different between the WT and Lamb2^−/−^, the LI_[0.5]_ (LI in mice that received a single EdU injection 30 min prior to tissue harvest) was significantly reduced in the *Lamb2* ^*−/−*^ retinas. This suggests that there is a decreased proportion of RPCs in S-phase at a given time in *Lamb2* ^*−/−*^ retinas. Calculation of T_C_ and T_S_ resulted in values of 42.6 and 24.4 h for WT, and 29.3 and 11.5 h for *Lamb2* ^*−/−*^ retinas, respectively. The calculated WT values are similar to the ones previously reported (Alexiades and Cepko, 1996). These data suggest that *Lamb2* deletion results in shortening of the S-phase and the cell cycle in general. It is noteworthy that a previous study demonstrated S-phase shortened in RG and IPCs undergoing neurogenic divisions, compared to proliferative divisions (Arai et al., 2011). Together, these data are consistent with our previous report that *Lamb2* deletion results in a shift of multipotent RPCs towards fate-restricted rod progenitors (Serjanov et al., 2018).

### Exogenous Laminin β2 Rescues RPC Cell Cycle and S-phase Timing

To confirm our findings and to test whether β2-containing laminins at the ILM directly affect RPC cell cycle dynamics, we performed cumulative EdU labeling studies *ex vivo*. Retinal explants were prepared from P0 eyes, and grown in the top compartment of transwells, with the retinal pigmented epithelium (RPE) intact, ganglion cell layer up. After 1 day *in vitro* (DIV), a droplet of medium containing recombinant laminin 521 (trimer containing α-5, β-2, and γ-1 chains) was added to the retinal surface. In so doing, laminin β2 is introduced into the retina as a functional trimer. Medium without recombinant laminin was used as a control. Rescue by exogeneous addition of laminin *in vitro* has been previously used to great success (Li et al., 2002; Gnanaguru et al., 2013; Serjanov et al., 2018). Following that, culture medium containing 2 µM EdU was used to replace half the volume of the bottom compartment, as well as added to the top compartment at times ranging from 0.5 to 21.5 h prior to tissue fixation at 3DIV. Analysis of the resulting saturation curves revealed a decrease in GF in *Lamb2* ^*−/−*^ explants compared to the WT. Addition of laminin 521 to the surface of the *Lamb2* ^*−/−*^ cultures rescued this phenotype (Figure 1E). Calculation of T_C_ and T_S_ resulted in values of 36.5 and 16.0 for WT; 30.1 and 10.3 for *Lamb2* ^*−/−*^; and 37.4 and 16.3 h for *Lamb2* ^*−/−*^ +521, respectively. Together, these data demonstrate that the presence of β2-containing laminins at the retinal surface is necessary for proper timing of the cell cycle and the S-phase.

### RPC G2/M Progression Is Laminin-dependent

**FIGURE 2:**
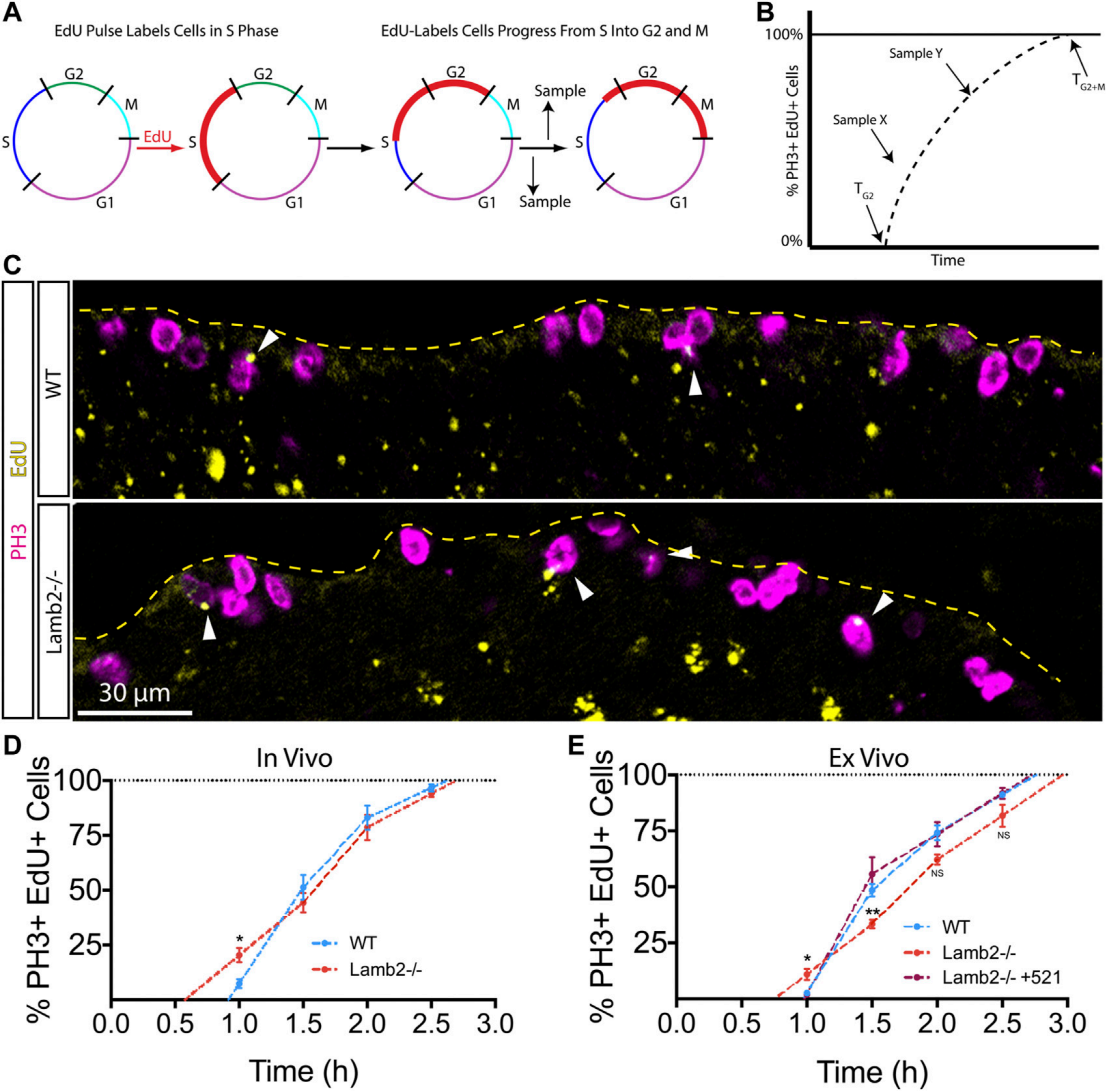
RPC G2/M progression is laminin-dependent. **(A)**. Model representation of the principles of the percentage of labeled mitosis method. This procedure relies on a brief EdU exposure of proliferating cells. Tracking the dynamics of mitotic entry of EdU+ cells (red), allows determining the time it takes to go from S to M-phase. **(B)**. Model illustrating the quantification of the labeled mitosis experiments. The time of G2 (T_G2_) is determined as the time needed for EdU+ cells to enter mitosis (become PH3+). Time of mitosis (T_M_) is calculated as the time required for EdU+/PH3+ population to replace EdU-/PH3+ population. **(C)**. Representative images of labeled mitosis experiments performed at P3, and collected 1 h after EdU injection. Dashed line indicates the apical surface of the retina. Arrowheads indicate EdU+/PH3+ cells, marking RPCs that have gone from S, into M-phase within 1 h. **(D)**. *In vivo* percentage of labeled mitosis saturation graphs of P3 retinas. Lamb2^−/−^ retinas present faster rate of M-phase entry and delayed initial mitotic progression, compared to WT. **(E).** *Ex vivo* percentage of labeled mitosis saturation graphs of retinal explants. Lamb2^−/−^ explants present faster rate of M-phase entry and delayed initial mitotic progression, similar to *in vivo* results. Each data point represents the average of technical and biological experimental replicates ±SEM. Statistical analysis was performed using Student’s t-test. *—*p* ≤ 0.05. **—*p* ≤ 0.01. NS—not significant.

To further investigate the effects of β2-containing laminins on RPC cell cycle dynamics, we examined the timing of G2 and M phases using the percent of labeled mitoses approach (Quastler and Sherman, 1959). To examine the G2/M dynamics, P3 mice were injected with EdU, and retinas were collected at 1, 1.5, 2, and 2.5 h intervals to assess the EdU saturation of mitotic cells labeled with anti-phospho-Histone H3^Ser28^ (PH3) antibodies. (Figure 2A). Observing the dynamics of EdU saturation of the PH3+ population allowed calculation of lengths of G2 (T_G2_), as determined by the time needed for EdU+ cells to become PH3+, reflecting time needed for cells to go from S to M phases; and M (T_M_), as quantified by the time between EdU+ cells becoming PH3+ and all PH3+ cells becoming EdU+, reflecting the time needed for EdU + cells to replace all EdU- mitotic cells (Figure 2B). Inspection of PH3+ RPCs revealed an increased number of mitotic EdU + RPCs 1 hour after EdU injection in *Lamb2* ^*−/−*^ retinas relative to the WT (Figure 2C). Analysis of later time points revealed a slower initial rate of EdU saturation in Lamb2^−/−^ retinas, as determined by comparing the slopes of the lines connecting the one and 1.5 h time points, while the later phase was unaffected (Figure 2D). Our WT EdU+/PH3+ saturation values closely resemble those reported previously (Pacal and Bremner, 2012). Analysis of the mitotic EdU saturation dynamics allowed calculation of T_G2_ and T_M_, which were 0.9 and 1.7 h for WT; and 0.6 and 2.1 h for *Lamb2* ^*−/−*^, respectively. These data suggest that *Lamb2* deletion results in accelerated G2 and prolonged M in the RPCs.

### Exogenous Laminin β2 Rescues RPC G2 and M Phase Progression

To confirm our findings and to test whether β2-containing laminins at the ILM directly affect RPC G2/M progression, we performed the percent of labeled mitosis studies *ex vivo*. Retinal explants were prepared as described above, but the EdU-containing medium was added to the top chamber of the transwells containing the retinal explants at 1, 1.5, 2, or 2.5 h prior to tissue collection. Similar to our *in vivo* findings, *Lamb2* ^*−/−*^ cultures displayed accelerated G2 and delayed M progression, with slower initial EdU+/PH3+ saturation rate as compared to WT explants. Addition of laminin 521 to the surface of *Lamb2* ^*−/−*^ retinal explants rescued G2/M dynamics (Figure 2E). Analysis of the mitotic EdU saturation revealed T_G2_ and T_M_ to be 1.0 and 1.8 h for WT; 0.8 and 2.2 h for Lamb2^−/−^; and 1.0 and 1.7 h for Lamb2^−/−^ +521 cultures, respectively. Together these data demonstrate that the presence of β2-containing laminins at the retinal surface is necessary for proper timing of the G2 and M phases in RPCs.

### β2-Containing Laminins Modulates RPC Cell Cycle Dynamics via Dystroglycan

**FIGURE 3:**
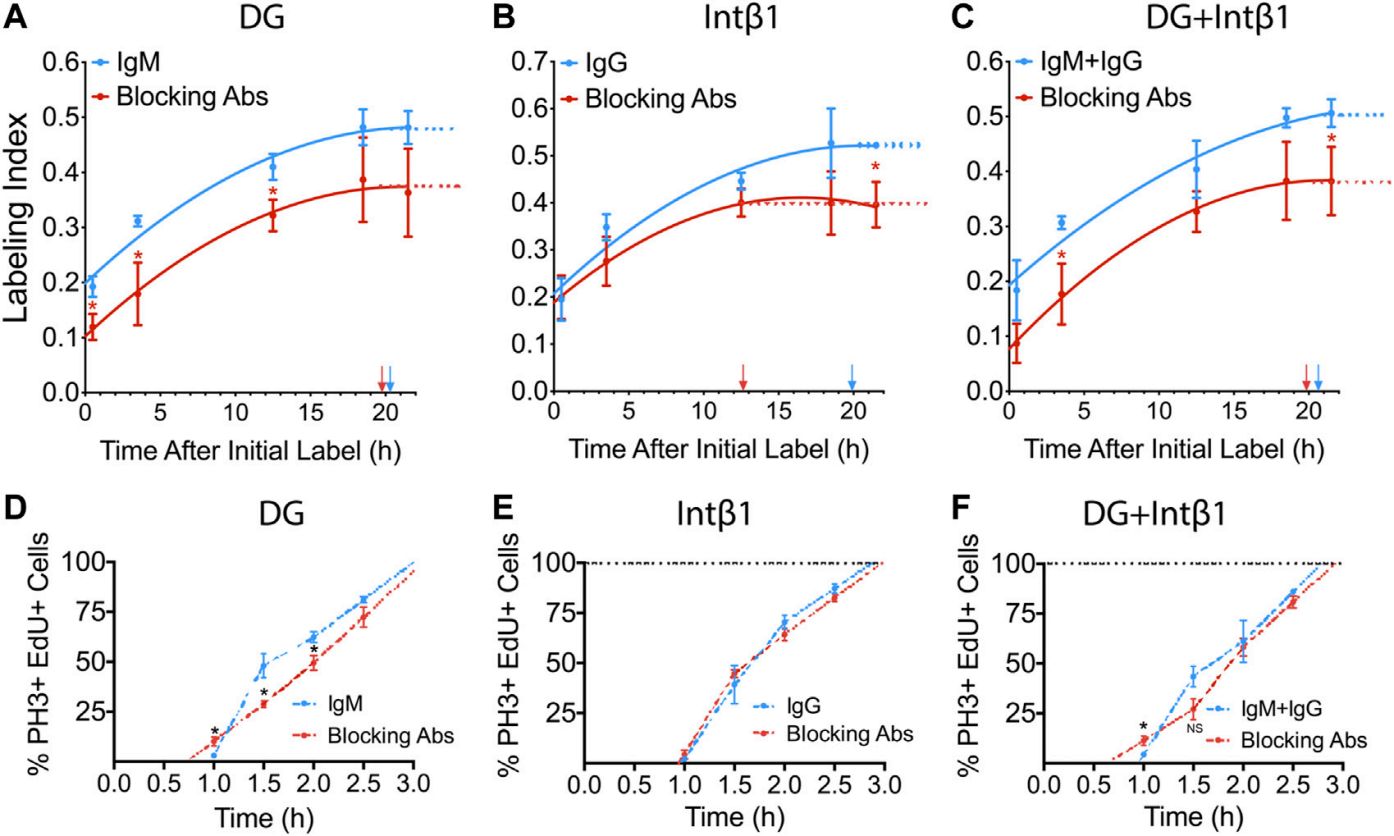
DG and intB1 regulate RPC cell cycle dynamics. **(A)**. Cumulative S-phase EdU labeling graphs of WT retinal explants with a-DG blocking antibodies. **(B)**. Cumulative S-phase EdU labeling graphs of WT retinal explants with intβ1 blocking antibodies. **(C)**. Cumulative S-phase EdU labeling graphs of WT retinal explants with α-DG and intβ1 blocking antibodies. Arrows indicate T_C_-T_S_. **(D)**. Labeled mitoses graphs for WT retinal explants with α-DG blocking antibodies. **(E).** Labeled mitoses graphs for WT retinal explants with intβ1 blocking antibodies. **(F)**. Labeled mitoses graphs for WT retinal explants with a-DG and intB1 blocking antibodies. Each data point represents the average of technical and biological experimental replicates ±SEM. Statistical analysis was performed using Student’s t-test. *—*p* ≤ 0.05. NS—not significant.

We have previously reported that *Lamb2* deletion results in RPC basal process retraction, leading to disruption of ECM-RPC contact and mislocalization of its receptors—DG and intβ1, and that their proper localization is restored by addition of laminin 521 *ex vivo* (Serjanov et al., 2018). Thus, we proceeded to investigate the role of these receptors in transducing the signals that regulate the cell cycle progression, from the ECM to the RPCs. To do so, we performed a series of *ex vivo* cumulative EdU labeling experiments. WT retinal cultures were prepared as described above, with an additional step of applying either α-DG (IIH6) or intβ1 (9EG7) function-blocking antibodies to the retinal surface. Blocking α-DG signaling resulted in reduction of both LI_[0.5]_ as well as the GF, relative to control antibodies (Figure 3A), and similar to the *Lamb2* ^*−/−*^ (Figure 1E). The resulting T_C_ and T_S_ values were 34.6 and 14.3 h for the control antibody cultures, and 27.2 and 7.5 h for the α-DG blocking cultures, respectively. Blocking intβ1 signaling did not affect LI_[0.5]_, but decreased the GF as well as the time needed to reach the saturation plateau (Figure 3B). The resulting T_C_ and T_S_ values were 33.0 and 13.1 h for the control antibody cultures, and 23.9 and 11.3 h for the intβ1 blocking cultures, respectively. Blocking both receptors resulted in a curve similar to one obtained from α-DG, without intβ1-block features (Figure 3C). The resulting T_C_ and T_S_ values were 33.5 and 12.9 h for the control antibody cultures, and 24.8 and 5.0 h for the compound blocking cultures, respectively. These data suggest that the shortening of T_C_ and T_S_ observed in *Lamb2* ^*−/−*^ RPCs are due to impaired DG-mediated signaling.

To further assess the roles of the laminin receptors in controlling cell cycle dynamics, we performed a series of labeled mitoses studies in retinal explants that were treated with α-DG or intβ1 blocking antibodies. Similar to the mitosis labeling dynamics observed in *Lamb2* ^*−/−*^ cultures (Figure 2E), α-DG blocking resulted in accelerated mitotic entry, and slower initial progression through mitosis (Figure 3D). Analysis of the mitotic EdU saturation revealed T_G2_ and T_M_ to be 1.0 and 2.0 h in control cultures, and 0.7 and 2.4 h in α-DG block cultures, respectively. Intβ1 blocking did not have an effect on mitosis labeling relative to the control (Figure 3E). Analysis of the mitotic EdU saturation revealed T_G2_ and T_M_ to be 0.97 and 1.9 h in control cultures, and 0.95 and 2.0 h in intβ1 blocked cultures, respectively. Compound block of both α-DG and intβ1 resulted in accelerated mitotic entry and delayed initial progression, similar to blocking α-DG alone (Figure 3F). Analysis of the mitotic EdU saturation revealed T_G2_ and T_M_ to be 0.9 and 1.8 h for the control cultures, and 0.6 and 2.3 h for the compound block cultures, respectively. Together these data demonstrate that DG-mediated signaling controls cell cycle dynamics in RPCs, independently of intβ1.

Combining all the data from both *in vivo* and *ex vivo* studies, we were able to calculate the lengths of G1 (T_G1_) for each condition by simple arithmetic: *T*_*G1*_ *= T*_*C*_ *-T*_*S*_ *-T*_*G2*_ *-T*_*M*_. The resulting T_G1_ values reveal no effect on T_G1_ in *Lamb2* ^*−/−*^, or α-DG blocked conditions, while showing that it was considerably decreased in intβ1 blocked conditions. Complete cell cycle dynamics are summarized in Table 1. Taken together, these data suggest that β2-containing laminins regulate RPC cell cycle progression through DG-mediated signaling.

**TABLE 1:** *In vivo* and *ex vivo* RPC cell cycle parameters.

	***In Vivo***						
	T_C_	T_G1_	T_S_	T_G2_	T_M_	GF±SD	T_C_-T_S_
WT	42.6	15.5	24.4	0.9	1.7	37.9 ± 1.7%	18.2
Lamb2^−/−^	29.3	15.2	11.5	0.6	2.1	39.6 ± 2.1%	17.9

	***Ex Vivo***						
	T_C_	T_G1_	T_S_	T_G2_	T_M_	GF±SD	T_C_-T_S_
WT	36.5	17.8	16.0	1.0	1.8	52.6 ± 4.2%	20.6
Lamb2^−/−^	30.1	16.9	10.3	0.8	2.2	39.3 ± 3.4%	19.9
Lamb2^−/−^ +521	37.4	18.4	16.3	1.0	1.7	48.9 ± 1.8%	21.1
Control IgM	34.6	17.5	14.3	1.0	2.0	48.0 ± 2.8%	20.3
α-DG	27.2	16.8	7.5	0.7	2.4	37.3 ± 6.9%	19.7
Control IgG	33.0	17.1	13.1	0.97	1.9	52.1 ± 4.7%	19.9
IntB1	23.9	9.6	11.3	0.95	2.0	39.9 ± 5.2%	12.6
Control IgM + IgG	33.5	17.8	12.9	0.9	1.8	50.2 ± 2.0%	20.6
α-DG + IntB1	24.8	16.8	5.0	0.6	2.3	38.3 ± 5.7%	19.8

Cell cycle parameters were calculated from the data in Figures 1D,E, 2D,E, 3A–F. Times shown in hours. T_C_, time of cell cycle, T_G1_, time of G1, T_S_, time of S, T_G2_, time of G2, T_M_, time of M.

### RPC S-phase Progression Is Laminin-dependent by DG Pathway

**FIGURE 4:**
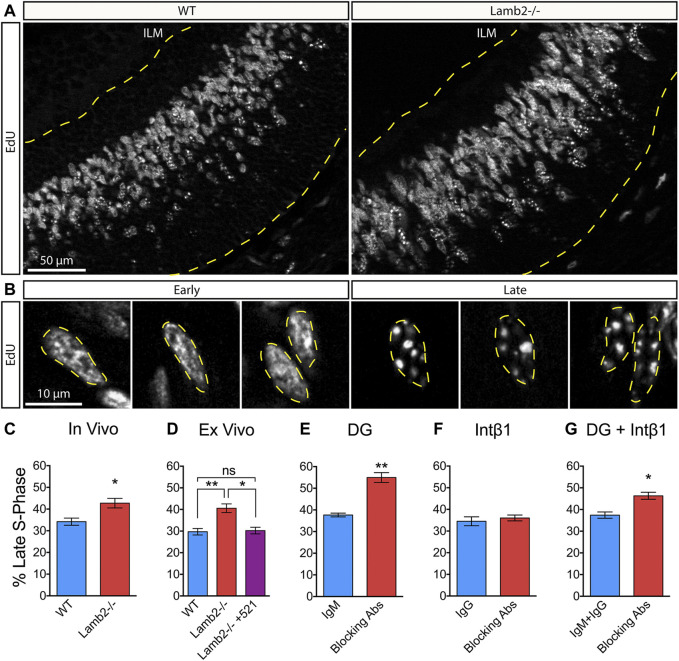
β2-containing laminins regulate RPC S-phase progression via DG. **(A)**. EdU labeling in P3 retinal cross-sections. Dashed lines delineate the retinal tissue limits. ILM—inner limiting membrane. **(B)**. High power representative images of RPCs in early (left) and late (right) S-phases. Replicon activity is reflected in pattern of EdU incorporation. Early S-phase is characterized by high number of active replicons, leading to a uniform EdU incorporation. Late S-phase is characterized by low number of active replicons, leading to punctate EdU incorporation. **(C)**. Quantification of RPCs in late S-phase relative to RPCs in all stages of S-phase in P3 WT and Lamb2^−/−^ cross-sections. Lamb2^−/−^ RPCs display higher percentage of cells in late S-phase relative to WT. **(D)**. Quantification of RPCs in late S-phase in retinal explants. Lamb2^−/−^ explants display higher percentage of late S-phase cells, compared to WT. Addition of laminin 521 rescues this effect, restoring normal early/late S-phase ratios. **(E)**. Quantification of RPCs in late S-phase in WT retinal explants with and without α-DG blocking antibodies. Blocking α-DG signaling results in increased percentage of late S-phase RPCs. **(F)**. Quantification of RPCs in late S-phase in WT retinal explants with and without intβ1 blocking antibodies. Blocking intβ1 signaling has no effect on percentage of late S-phase RPCs. **(G)**. Quantification of RPCs in late S-phase in WT retinal explants with and without a combination of α-DG and intβ1 blocking antibodies. Blocking both signaling pathways results in increased percentage of late S-phase RPCs. Each data point represents the average of technical and biological experimental replicates ± SEM. Statistical analysis was performed using Student’s t-test. *—*p* ≤ 0.05. **—*p* ≤ 0.01. NS—not significant.

Having observed a shortening of the S-phase in *Lamb2* ^*−/−*^ retinas, we proceeded to further investigate the effects of β2-containing laminins on dynamics of the S-phase progression. Eukaryotic nuclei contain over 10^4^ replication domains (Hand, 1978). In early S-phase, hundreds of these domains are active and distributed throughout the nucleoplasm, while only tens are active in late S-phase (Manders et al., 1992; 1996). This allows for an easy identification of cells in early vs late stages. Thymidine analogue labeling of newly synthesized DNA of cells in early S-phase appears as largely uniform staining, composed of hundreds of small labeled domains scattered throughout the nucleoplasm, while late S-phase replicons appear much larger in size and fewer in number, both *in vitro* as well as *in vivo* (Manders et al., 1992; Manders et al., 1996; Jaunin et al., 1998; Ma et al., 1998; Yamada et al., 2005). We used this cytological feature to assess whether *Lamb2* deletion affects RPC S-phase progression in addition to duration. P3 retinas were collected 1 hour after a single EdU injection, and analyzed in cross sections (Figure 4A). Consistent with the literature, RPC nuclei in early and late S-phases were easily discernable. EdU labeling of early S-phase nuclei was largely uniform throughout the nucleoplasm, while the late S-phase nuclei presented a small number of large EdU+ puncta (Figure 4B). Additionally, the positioning of the EdU+ RPCs was consistent with the interkinetic nuclear migration, where early S-phase cells were located basally, while the late S-phase cells were located apically (Figure 4A). Analysis of the EdU labeling revealed a significant increase in late S-phase RPCs in *Lamb2* ^*−/−*^ retinas (Figure 4C). These findings suggest that *Lamb2* deletion causes an increase in RPCs residing in late stages of S-phase.

To test whether the observed increase in late S-phase RPCs is directly due to the loss of β2-containing laminins at the retinal surface, we performed *ex vivo* rescue studies, and analyzed early to late S-phase ratios. As culture system cannot clear EdU, medium containing 2 µm EdU was added to the top transwell compartment 30 min prior to fixation, rather than 1 h, as was done *in vivo,* to prevent continuous labeling, which may alter the results. Similar to the *in vivo* results, the percentage of late S-phase RPCs was significantly increased in *Lamb2* ^*−/−*^ explants compared to WT. Addition of laminin 521 rescued this effect and restored the early-to-late S-phase ratios to WT levels (Figure 4D). Together, these data demonstrate that β2-containing laminins at the retinas surface directly affect RPC S-phase progression.

Following these results, we proceeded to investigate whether DG was responsible for mediating the ECM-RPC signaling that regulates the S-phase progression, in addition to duration. As α-DG blocking phenocopies the *Lamb2* ^*−/−*^ cell cycle dynamics (Table 1), we hypothesized that it would also affect the S-phase dynamics in the same way *Lamb2* deletion does. Indeed, blocking α-DG in retinal explants resulted in a significant increase of late S-phase RPCs (Figure 4E), while intβ1 blocking had no effect (Figure 4F). Compound blocking of both receptors resulted in an increase of late S-phase RPCs as well, though not as pronounced as α-DG block alone (Figure 4G). Taken together, these data demonstrate the RPC S-phase dynamics are laminin-dependent and regulated by DG.

### RPC Mitosis Progression Is Laminin-dependent and Modulated by DG

**FIGURE 5:**
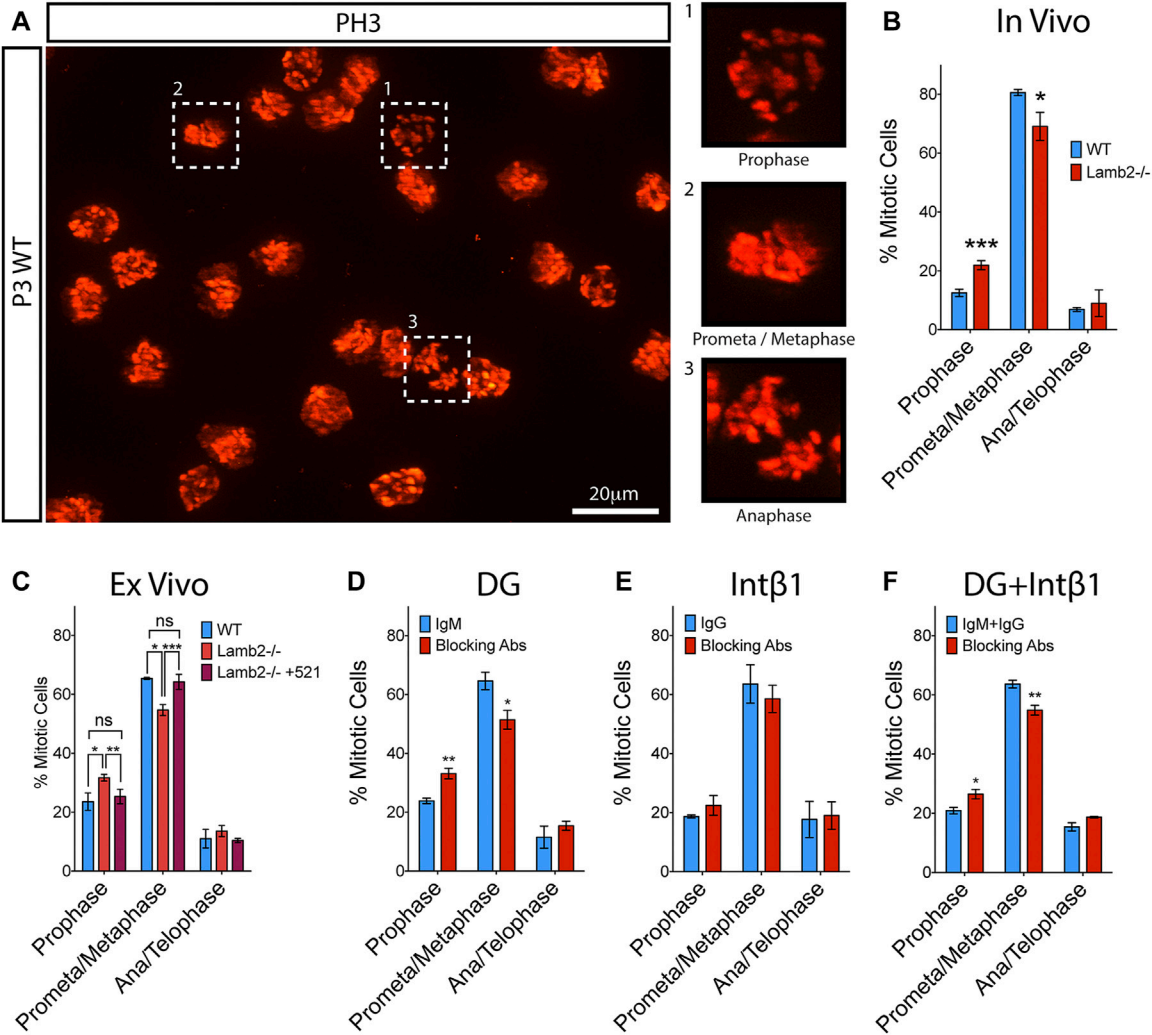
β2-containing laminins regulate RPC M-phase progression via DG. **(A)**. Extended focus view of a z stack obtained from the apical surface of a retinal flat-mount. As PH3 is associated with condensed chromatin, it allows for mitotic staging, based on its staining pattern within the cell. **(B)**. Quantification of RPCs in various stages of mitosis in WT and Lamb2^−/−^ retinas. Lamb2^−/−^ retinas display higher relative percentages of RPCs in prophase with a concomitant decrease of ones in prometa/metaphase, compared to WT. **(C)**. Quantification of RPCs in various stages of mitosis in WT and Lamb2^−/−^ retinal explants. Lamb2^−/−^ explants display higher relative percentages of RPCs in prophase with a concomitant decrease of ones in prometa/metaphase, similar to *in vivo* results. Addition of laminin 521 to Lamb2^−/−^ explants rescues normal mitosis stage ratios. **(D)**. Quantification of RPCs in various stages of mitosis in WT retinal explants with and without α-DG blocking antibodies. Blocking α-DG signaling results in higher relative percentages of RPCs in prophase with a concomitant decrease of ones in prometa/metaphase, relative to control. **(E)**. Quantification of RPCs in various stages of mitosis in WT retinal explants with and without intB1 blocking antibodies. Blocking intβ1 signaling has no effect on mitotic stage distribution of RPCs. **(F)**. Quantification of RPCs in various stages of mitosis in WT retinal explants with and without a compound α-DG/intβ1 blockade. Blocking both signaling pathways results in higher relative percentages of RPCs in prophase with a concomitant decrease of ones in prometa/metaphase, similar to Lamb2^−/−^ and WT+α-DG block. Each data point represents the average of technical and biological experimental replicates ±SEM. Statistical analysis was performed using Student’s t-test. *—*p* ≤ 0.05. **—*p* ≤ 0.01. ***—*p* ≤ 0.001. NS—not significant.

Cell fate choice of the RG in the developing cortex is known to be affected by the length of mitosis as well as its progression dynamics. Previous study reported that cells dwelling for an extended period in prometaphase have an increased propensity to produce postmitotic or apoptotic daughter cells (Pilaz et al., 2016). We have observed an extended M-phase duration (Table 1) and an apparent initial delay in mitotic progression in *Lamb2* ^*−/−*^ RPCs (Figures 2D,E). We, therefore, proceeded to investigate whether the M-phase dynamics are affected by β2-containing laminins. Mitotic RPCs were visualized in retinal flat-mounts using PH3. Mitosis phases were inferred from the PH3 staining pattern obtained from z-stacks of the retinal apical surface (Figure 5A). As Histone H3 phosphorylation is associated with chromosome condensation and segregation (Rossetto et al., 2012), PH3 staining provides a useful tool in determining the mitotic state of the cell. In prophase, when chromosomes begin to condense, PH3 appears discontinuous and punctate, reflecting the state of chromatin condensation (Figures 5A–1). In prometaphase, the chromosomes become fully condensed, and PH3 labels the chromosomes entirely. The chromosomes then align at the metaphase plate during metaphase (Figures 5A–2). At anaphase, the chromosomes segregate towards the opposing mitotic spindle poles (Figures 5A–3). During late anaphase, Histone H3 becomes dephosphorylated by PP1 due to chromosome decondensation, and can be observed in late anaphase/telophase as faint staining surrounding the chromosomes (not shown). Analysis of the mitotic RPCs in P3 retinas revealed a significant increase in the number of cells in prophase with a concomitant significant decrease in the prometa/metaphase population, in *Lamb2* ^*−/−*^ retinas. The late-M population was unaffected (Figure 5B). These data are consistent with EdU/PH3 saturation dynamics, where the initial mitosis progression is significantly slower, while the late stage is unaffected (Figure 2D).

To confirm that the prophase extension is directly affected by β2-containing laminins at the retinal surface, we performed *ex vivo* rescue studies. Analysis of the flat-mounted retinal explants revealed a significant increase in prophase and a significant decrease in prometa/metaphase, without affecting the late-M populations, in *Lamb2* ^*−/−*^ cultures. Addition of laminin 521 to *Lamb2* ^*−/−*^ retinas restored the normal M-phase dynamics. These data are consistent with the EdU/PH3 saturation dynamics described above (Figure 2E). Together these data demonstrate the direct link between the β2-containing laminins in the ECM and mitosis dynamics in the RPCs.

To further investigate the molecular mechanisms governing the regulation of RPC mitosis dynamics, we performed a series of *ex vivo* receptor blocking studies to elucidate the roles of laminin receptors in this pathway. α-DG blocking resulted in a significant increase in the prophase population, with a concomitant decrease in prometa/metaphase population. The late-M population was not affected (Figure 5D). Intβ1 blocking did not change mitosis dynamics (Figure 5E). Compount α-DG and intβ1 blocking resulted in a significant increase in the prophase population, with a concomitant decrease in prometa/metaphase population, similar to α-DG-only block. The late-M population was not affected (Figure 5F). These data are in agreement with the results of EdU/PH3 saturation studies described above (Figures 3D–F). Together, these data demonstrate that the M-phase dynamics are laminin-dependent, and mediated by the DG signaling pathway.

### RPC Cell Cycle Re-entry Is Laminin Dependent

**FIGURE 6:**
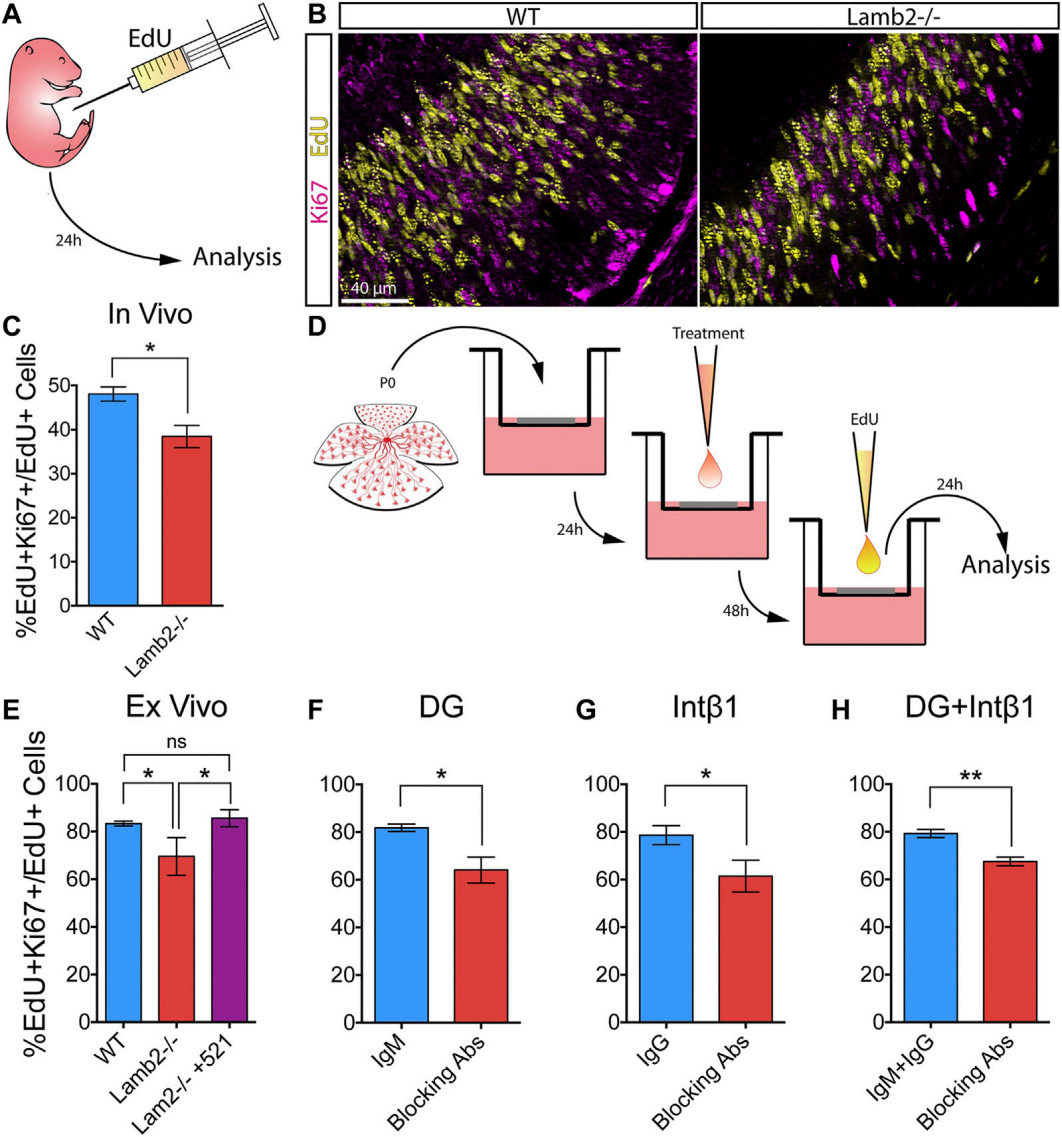
β2-containing laminins regulate RPC cell cycle re-entry via DG and intβ1. **(A)**. Experimental paradigm for determining the rate of RPC cell cycle reentry. P3 mice were administered a single intraperitoneal EdU injection, and their retinas were collected 24 h later. The resultant retinal cross-sections were then stained for EdU (proliferating RPCs at P3) and Ki67 (proliferating RPCs at P4) to assess the rate of RPC cell cycle re-entry. **(B)**. Representative images of P4 retinal cross-sections stained for EdU and Ki67. **(C)**. Quantification of EdU/Ki67 stained retinas. EdU+Ki67 + RPCs (RPCs that have re-entered the cell cycle) were significantly decreased in Lamb2^−/−^ retinas, compared to WT. **(D)**. Experimental paradigm for determining the rate of RPC cell cycle reentry in retinal explants. Explants were prepared from P0 retinas and grown ganglion cell layer up in transwells. After 24 h, a drop of medium containing either laminin 521, function-blocking antibodies, or control antibodies, was added to the retinal surface. After 48h, medium containing EdU was added to the top compartment, and the retinas were collected 24 h later. The resultant cross-sections were then stained for EdU and Ki67. **(E)**. Quantification of EdU/Ki67 stained retinal explants. EdU+Ki67 + RPCs (RPCs that have re-entered the cell cycle) were significantly decreased in Lamb2^−/−^ explants, compared to WT. Addition of laminin 521 to Lamb2^−/−^ explants restored normal cell cycle re-entry. **(F)**. Blocking α-DG signaling in WT retinal explants significantly reduced the rate of RPC cell cycle re-entry. **(G)**. Blocking intβ1 signaling in WT retinal explants significantly reduced the rate of RPC cell cycle re-entry. **(H)**. Blocking both α-DG and intβ1 signaling in WT retinal explants significantly reduced the rate of RPC cell cycle re-entry. Each data point represents the average of technical and biological experimental replicates ±SEM. Statistical analysis was performed using Student’s t-test. *—*p* ≤ 0.05. **—*p* ≤ 0.01. NS—not significant.

Having observed altered cell cycle dynamics in *Lamb2* ^*−/−*^ retinas, we proceeded to investigate whether the observed changes affected the RPC self-renewal. P3 mice were administered a single intraperitoneal EdU injection, and the retinas were collected 24 h later (Figure 6A). Retinal cross-sections were then stained for EdU and Ki67, to detect RPCs that were proliferating at P3 and those proliferating at P4, respectively (Figure 6B). As Ki67 is expressed from late G1 to the end of M (Pacal and Bremner, 2012), it provides a useful tool for discriminating between EdU+ cells that have re-entered, or exited the cell cycle. Analysis of the percentage of EdU+ cells that were also Ki67+ revealed a significant decrease of the double-labeled RPC population in *Lamb2* ^*−/−*^ retinas compared to WT (Figure 6C).

To confirm that increased RPC cell cycle exit is the direct result of lack of β2-containing laminins at the retinal surface, we performed a series of *ex vivo* rescue studies. Medium containing 2 µM EdU was added to the top transwell compartment of 3DIV retinal explants, and the tissues were collected 24 h later, at 4DIV (Figure 6D). Similar to the *in vivo* results, *Lamb2* ^*−/−*^ explants exhibited a significant decrease of the EdU+Ki67+ population compared to WT. Addition of laminin 521 rescued this effect (Figure 6E). Together, these results demonstrate that RPC cell cycle re-entry is directly affected by β2-containing laminins at the retinal surface.

### DG and intβ1 Modulate RPC Cell Cycle Re-entry

Having established the role of β2-containing laminins in regulating RPC cell cycle re-entry and exit, we proceeded to examine the roles of DG and intβ1 in mediating this effect. Using the *ex vivo* approach, we assessed RPC cell cycle re-entry following receptor blockade. Blocking either α-DG or intβ1 resulted in a significant decrease of the EdU+/Ki67+ population compared to control (Figures 6F,G). Combining the two treatments also resulted in a significant decrease in RPC cell cycle re-entry. These effects did not appear to be additive, as compound blocking of both receptors did not result in a significantly greater effect than either receptor blocking alone (Figure 6H). These data suggest that DG and intβ1 mediated signaling pathways are involved in the regulation of RPC proliferation in rather complex fashion (see discussion for further comments).

## Discussion

**FIGURE 7:**
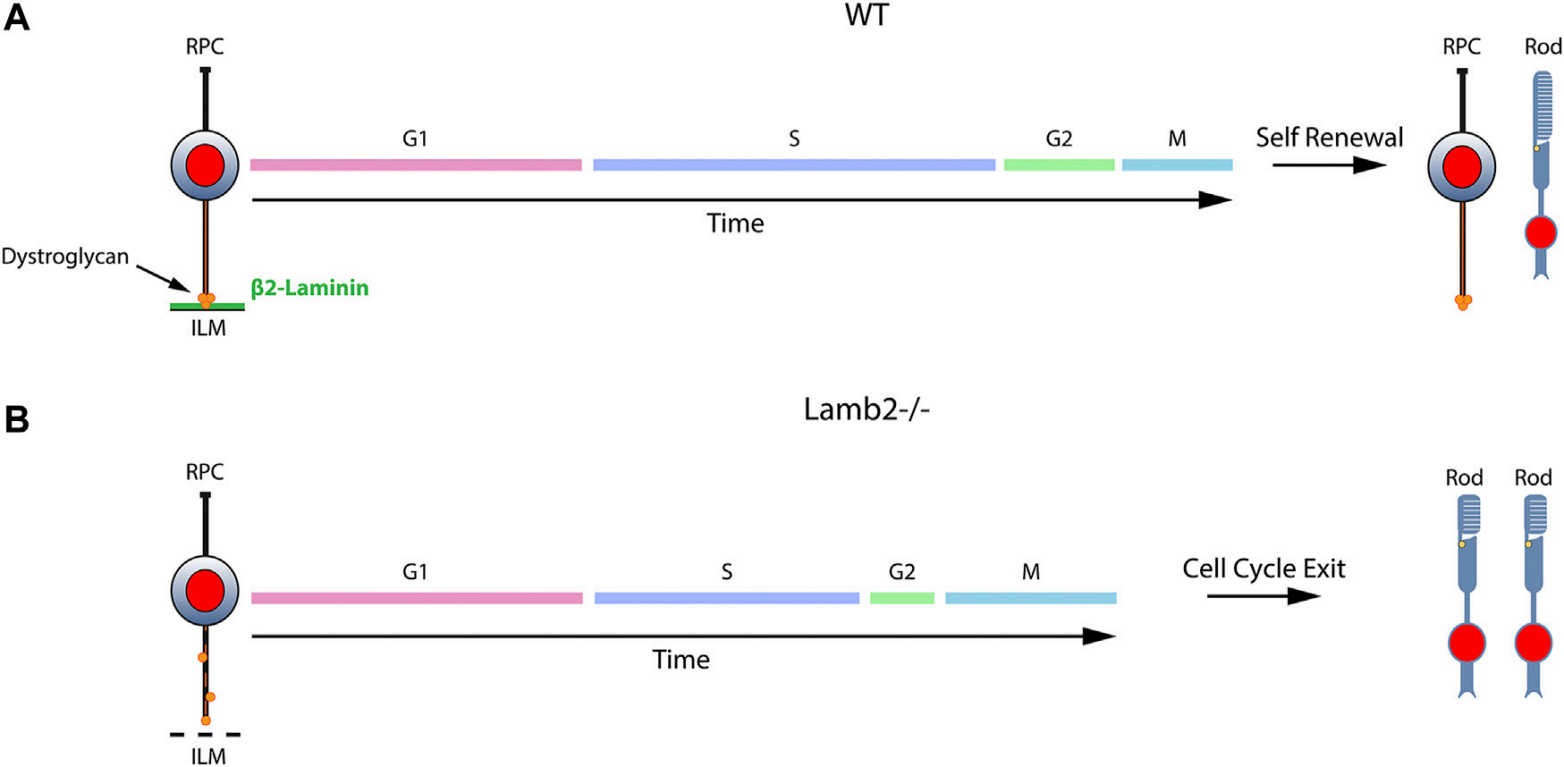
β2-containing laminins govern RPC proliferation by modulating the cell cycle dynamics via DG. Contact between RPCs and the ILM is maintained throughout the cell cycle via a variety of receptors. The signaling cascades modulated by these receptors play important roles in controlling the cell cycle dynamics and the ensuing cell fate. **(A)**. DG mediates the signaling between RPCs and β2-containing laminins in the ILM. Cells progress through cell cycle and proceed to divide in a self-renewing fashion, maintaining proper progenitor pool, or exit the cell cycle to produce retinal neurons. **(B)**. Loss of laminin β2 leads to disrupted ILM, which fails to provide proper binding sites to DG. This leads to mislocalization of the receptor and alters its molecular signaling pathways. As a result, RPC cell cycle dynamics are altered, and the resulting mitoses lead to premature cell cycle exit with overproduction of rods and premature progenitor pool depletion.

Recent progress in understanding of the ECM functions in development has greatly expanded our appreciation of the importance of the complex microenvironment in which developmental processes take place. While ECM has been shown to play important roles in multiple processes on both cellular and tissue levels, its effects on cell cycle dynamics have remained largely unexplored. Here, we have identified the molecular signaling mechanism by which β2-containing laminins regulate RPC cell cycle dynamics and, as a result, the choice between RPCs producing proliferating or post-mitotic progeny. We have established that 1) laminin-dependent signaling is involved in the regulation of the RPC cell cycle dynamics; 2) DG-mediated signaling is responsible for mediating the laminin-RPC signaling responsible for control of the cell cycle dynamics; 3) laminin-DG signaling is responsible for proper S-phase progression; 4) laminin-DG signaling is responsible for proper M-phase progression. Figure 7 presents a schematic summary of these findings. The role of ECM in modulating RPC cell cycle dynamics has wide reaching implications not only in the field of developmental biology, but in pathobiology as well, shedding light on basic cellular processes.

### Laminins Guide Proper RPC Cell Cycle Dynamics

Numerous studies have noted the existence of a relationship between the cell cycle dynamics and progenitor cell fate determination. The initial reports noted that lengthening of G1 is associated with increased cortical neurogenesis (Calegari, 2003; Calegari, 2005; Pilaz et al., 2009). Further studies of the neurogenesis dynamics revealed G1-extension to be specifically associated with the IPCs, which are biased towards more neurogenic than proliferative divisions (Arai et al., 2011). Additionally, both RG and IPCs undergo a notably shorter S-phase in the cell cycle preceding terminal division (Arai et al., 2011). More recent studies demonstrated detrimental effects of prolonged M-phase on progenitor self-renewal (Pilaz et al., 2016). While changes in cell cycle dynamics during tissue histogenesis are well known to have profound effects on differentiation, the underlying mechanisms regulating these changes remain to be determined. Our current findings suggest that laminins in the ILM provide essential signaling cues that regulate RPC cell cycle progression.

The effects of *Lamb2* deletion of cell cycle dynamics and rate of mitotic exit reveal the importance of proper 3D microenvironment in development and morphogenesis. Our analysis of the cell cycle in *Lamb2* ^*−/−*^ provides further insight into the relationship between cell cycle and neurogenesis in the retina. Consistent with previous reports, we observed a reduced S-phase (Arai et al., 2011), and prolonged mitosis durations (Pilaz et al., 2016), accompanied by increased rate of cell cycle exit in *Lamb2* ^*−/−*^ retinas. Prolonged M-phase has been observed in lissencephaly and microcephaly models (Pilaz et al., 2016; Bershteyn et al., 2017). These findings are consistent with studies performed in zebrafish microcephaly models. RPCs of *stil* and *odf2*-deficient zebrafish embryos display prometaphase progression defects, followed by cell cycle exit or apoptosis (Novorol et al., 2013). These findings correlate with reports that both *Lamb2-*and DG-deficient mice also present cortical dysplasias (Moore et al., 2002; Satz et al., 2010; Radner et al., 2012).

Interestingly, our analysis of the prolonged M-phase dynamics differs from those reported previously. Reduced duration of prophase, and extended duration of prometa/metaphase has been observed in RG leaving cell cycle (Pilaz et al., 2016). We, on the other hand, observed increased numbers of RPCs in prophase, and reduction of those in prometa/metaphase in *Lamb2* ^*−/−*^ retinas. This difference in observations may be due to methodology. Studies describing prometaphase lengthening rely on live imaging of cortical slices, where mitosis stages are inferred from the appearance of the dividing cells. As such, live imaging in tissue is not reliable in distinguishing between late G2 and prophase. Our method, instead, relies on a molecular marker (PH3) that reflects the state of chromosome condensation, allowing for greater precision in determining the exact stage. Alternatively, it is possible that both observations are correct and describe differences in M-phase progression between different conditions and tissues. Accelerated G2, followed by prolonged prophase had been previously described in models with aberrant Cyclin A/CDK2 activity, and was proposed to be related to premature condensation of incompletely replicated DNA (Furuno et al., 1999). This raises the possibility that the overall length of mitosis, rather than that of its specific phases, is important in regulating cell fate. It has recently been proposed that cells with prolonged M-phase are deemed as “problematic” and removed from the cell cycle (Pilaz et al., 2016). Interestingly, altered prophase/prometaphase/metaphase dynamics have also been noted in cancer (Therman et al., 1984), suggesting that proper M-phase progression plays a role not only in development, but pathogenesis as well. Taken together with our current observations, all these findings suggest that prolonged mitosis is a common feature of limited progenitor self-renewal throughout the CNS both in development and pathology, and underlines the importance of DG-mediated ECM signaling in development.

### Distinct Roles of DG and intβ1 Signaling in Cell Cycle Regulation

We have previously reported that disruption of both DG and intβ1 signaling decreased RPC proliferation in a non-additive fashion (Serjanov et al., 2018). Consistent with those observations, our current study found that blocking α-DG or intβ1 in WT retinal explants decreased the progenitor pool and increased the rate of cell cycle exit, with the effects not appearing to be additive. Moreover, analysis of the cell cycle revealed completely different effects of either condition on its dynamics. While DG blocking results in shortening of S and G2-phases, with an extension of M, intβ1 blocking only shortened G1. Combination of both treatments mimics the cell cycle dynamics of DG block without any of the effects of intβ1 blockade. This suggests that the two receptors have distinct signaling pathways in regulation of the cell cycle, with DG being the main transducer of ECM-RPC signaling. This idea is corroborated by the fact that cell cycle dynamics of DG block phenocopy those of *Lamb2* ^*−/−*^ retinas, while intβ1 blocking does not.

Interestingly, while intβ1 blocking causes shortening of the G1-phase, there is still a significant increase in the rate of cell cycle exit. Previous studies noted that forced reduction of G1-phase duration by overexpression of cyclins D1 and E1 promoted cell cycle re-entry and reduced differentiation in the developing cortex (Pilaz et al., 2009). Our data suggest that the relationship between cell cycle dynamics and the ensuing cell cycle exit or re-entry decision is more complex than a straightforward length-dictates-fate scenario, and may be context-dependent. An alternative explanation could be that intβ1 blockade causes G1 arrest in a subpopulation of RPCs. As cumulative S-phase labeling method relies on cells continuously entering the S-phase, G1 arrest would prevent this from happening, thus making this population inaccessible to EdU label. This, however, is unlikely as LI_[0.5]_, which describes the ratio of cells in S-phase at any given time, is unaffected by intβ1 blockade, suggesting no defect in G1-S transition. Further investigation into the interplay between DG and intβ1, and their molecular signaling pathways in cell cycle regulation is required to shed more light on these processes.

It should be noted that there is a similarity between our observations of laminin-DG dependent regulation of the mitotic spindle (Serjanov et al., 2018) and the data presented here. Our observations of disrupted S-phase dynamics in DG-blocked retinal explants may in part explain the alterations in the behavior of the RPC centrosomes as centrosomal replication also occurs during the S-phase and disruptions of one have an effect on the other (Stearns, 2001; Sluder and Nordberg, 2004; Sluder, 2005; Kuriyama et al., 2007; Acilan and Saunders, 2008). Interestingly, in both cases ECM-intβ1 mediated signaling has a distinctly different effect on RPC proliferation and maintenance than does DG-mediated signaling. Also, in both cases of regulation of the mitotic spindle orientation as well as of the cell cycle, DG-mediated signaling appears to be the dominant pathway, as demonstrated by DG-block phenotype in DG+intβ1 retinal cultures. A similar dichotomy was seen between integrin and DG signaling in early embryonic development (Li et al., 2002).

### Implication of Laminin-DG Signaling in RPC Chromatin State Regulation

Shorter S-phase has been suggested to mean that differentiating cells spend less time error checking than in cells that need to produce more progenitors to ensure fidelity of the passed genetic material (Arai et al., 2011). Though this interpretation is logically sound, there may be deeper implications of this observation. *Lamb2* deletion, as well as blocking of α-DG, resulted in shorter S-phase, with a higher ratio of late vs early S-phase RPCs. The number and location of replicons differ between early and late S-phase (Manders et al., 1992; Manders et al., 1996), and reflect the higher order chromosome organization. During early S-phase, euchromatic regions are replicated, while the stable heterochromatin is replicated later (O'Keefe et al., 1992). Increased numbers of late S-phase RPCs in *Lamb2* ^*−/−*^ mice suggest higher heterochromatin content. This is consistent with an increase in rate of differentiation, as stem cells have largely euchromatic genomes, that become more transcriptionally restricted and condensed as they differentiate (Efroni et al., 2008; Cremer and Cremer, 2010; R. A.; Young, 2011). Whether chromatin condensation is the direct result of ECM-mediated signaling, or is secondary to disrupted proliferative cues remains to be determined. In either case, the role of ECM in regulating chromatin state as it pertains to neurogenesis offers a very interesting avenue of studies. Our data presented here suggest that laminins in the ILM regulate the chromatin state of the RPCs, which could in turn, affect the expression of multiple genes. As the role of the ECM composition in regulation of gene expression has been well established (Bissell et al., 1982; Maniotis et al., 1997; Kheradmand et al., 1998; Engler et al., 2006; Le Beyec et al., 2007), it is possible that deletion of *Lamb2* results in altered expression of signaling molecules that regulate the cell cycle progression and re-entry. The affected genes may include cell cycle regulators, various cytokines, and other ECM molecules that influence the stiffness of the ILM, which would affect the RPC cytoskeleton tension forces, as well as alter the biomolecular signaling properties of the surrounding ECM. Future studies would shed light on this phenomenon.

## Data Availability

The original contributions presented in the study are included in the article/Supplementary Material, further inquiries can be directed to the corresponding author.
